# Targeting DNA methylation can reduce cardiac injury associated with ischemia reperfusion: One step closer to clinical translation with blood-borne assessment

**DOI:** 10.3389/fcvm.2022.1021909

**Published:** 2022-09-28

**Authors:** Sri Rahavi Boovarahan, Abdullah F. AlAsmari, Nemat Ali, Rehan Khan, Gino A. Kurian

**Affiliations:** ^1^Vascular Biology Laboratory, School of Chemical and Biotechnology, SASTRA Deemed University, Thanjavur, India; ^2^Department of Pharmacology and Toxicology, College of Pharmacy, King Saud University, Riyadh, Saudi Arabia; ^3^Department of Pathology, Case Western Reserve University, Cleveland, OH, United States

**Keywords:** ischemia reperfusion injury, global DNA methylation, 5-azacytidine, mRNA expression, blood, myocardium

## Abstract

Ischemia reperfusion (I/R) injury is one of the main clinical challenges for cardiac surgeons. No effective strategies or therapy targeting the molecular and cellular mechanisms to reduce I/R exists to date, despite altered gene expression and cellular metabolism/physiology. We aimed to identify whether DNA methylation, an unexplored target, can be a potential site to curb I/R-associated cell death by using the left anterior descending artery occlusion model in male Wistar rats. I/R rat heart exhibited global DNA hypermethylation with a corresponding decline in the mitochondrial genes (PGC-1α, TFAM, POLG, ND1, ND3, ND4, Cyt B, COX1, and COX2), antioxidant genes (SOD2, catalase, and Gpx2) and elevation in apoptotic genes (Casp3, Casp7, and Casp9) expression with corresponding changes in their activity, resulting in injury. Targeting global DNA methylation in I/R hearts by using its inhibitor significantly reduced the I/R-associated infarct size by 45% and improved dysferlin levels via modulating the genes involved in cell death apoptotic pathway (Casp3, Casp7, and PARP), inflammation (IL-1β, TLR4, ICAM1, and MyD88), oxidative stress (SOD1, catalase, Gpx2, and NFkB) and mitochondrial function and its regulation (MT-ND1, ND3, COX1, ATP6, PGC1α, and TFAM) in the cardiac tissue. The corresponding improvement in the genes’ function was reflected in the respective hearts via the reduction in apoptotic TUNEL positive cells and ROS levels, thereby improving myocardial architecture (H&E staining), antioxidant enzymes (SOD, catalase activity) and mitochondrial electron transport chain activities and ATP levels. The analysis of blood from the I/R animals in the presence and absence of methylation inhibition exhibited a similar pattern of changes as that observed in the cardiac tissue with respect to global DNA methylation level and its enzymes (DNMT and TET) gene expression, where the blood cardiac injury markers enzymes like LDH and CK-MB were elevated along with declined tissue levels. Based on these observations, we concluded that targeting DNA methylation to reduce the level of DNA hypermethylation can be a promising approach in ameliorating I/R injury. Additionally, the blood-borne changes reflected I/R-associated myocardial tissue alteration, making it suitable to predict I/R-linked pathology.

## Introduction

Myocardial ischemia reperfusion (I/R) injury is an unavoidable pathology developed during revascularization procedures in ischemic heart patients (1). The cellular abnormalities of I/R get initiated with the imbalance in energy demand and supply of the tissue that induces irreversible biochemical changes and promotes the accumulation of metabolites (2). These escalated products alter the metabolic balance of the tissue via modifying the control of various genes involved in the biochemical pathways. In addition to the altered metabolism, I/R can induce an imbalance in calcium level, oxidant to antioxidant concentration, ionic balance of the cell and even pro to anti-inflammatory response (1). All these I/R-associated changes can alter the myocardium’s normal cellular niche, which in turn can influence the expression of certain sets of regulatory genes. Clinically, I/R pathology is manifested as myocardial stunning, hibernation, accelerated necrosis, and arrhythmia, which are the aftereffect of these cellular alterations (3).

Epigenetic modifications like DNA methylation and histone modifications play a key role in the underlying mechanisms involved in the pathology of cardiovascular diseases (CVD) (4). Unlike mutations, responses to epigenetic modifications are linked to the cellular environment that are influenced not only by the lifestyle of the subjects but also by the presence of co-existing diseases and therapeutics (5). Recent studies have shown that the pathology of many CVD are associated with hypermethylation of DNA and acetylation of histone proteins (4). Thus, targeting these epigenetic sites may be promising in managing I/R injury. However, in this direction, not much information is available with myocardial I/R injury, where the disruption of many cardiac genes’ expression is expected. Changed expression of genes, in turn, mediates a common final cascade of alterations wherein the heart reactivates numerous developmental pathways with a likely contribution from epigenetic modifications, especially DNA methylation (6). Global level and gene-specific DNA methylation patterns of the tissue, and blood as proxy markers have been widely studied in recent years to identify numerous disease conditions (7). However, gene-specific DNA methylation analysis cannot provide a holistic picture of all the DNA methylation changes within a genome at the global level.

Boovarahan and her group recently showed hypermethylation of DNA at the global level in the heart during I/R (8), and short term treatment with 5-azacytidine, an inhibitor of DNA methylation, can impart cardio-protection in *in vitro*, *ex vivo*, and *in vivo* models of I/R in rats by activation of the PI3K/GSK3β and mtK_*ATP*_ channel signaling axis (9). Aberrant DNA methylation has been reported in coronary heart diseases, where methylated genes of SMAD3, SERPINB9, and PRKCH are likely to represent novel biomarkers for the early diagnosis of the acute coronary syndrome (10). The cardiac cell contains spatially two distinct genomes, namely nuclear and mitochondrial, where previous studies have established that communications between these organelles coordinate the epigenetic process that regulates the response of cells to external cues (11).

Generally, methylation is tissue-specific, but recently, methylation patterns in the blood are frequently used as proxy tissue due to the difficulty in obtaining the tissue samples (12). Few investigators claimed that blood displays a highly distinctive methylomic profile from other somatic tissues (13). Little is known about whether the DNA methylation level measured in leukocytes can be used as an intermediate marker to assess I/R-associated cardiac abnormalities. Since the intermediate marker should be associated with the endpoint of interest, we need to compare the blood-linked changes with the heart and then to the injury. In the present study, we extensively evaluated the global DNA methylation level and its corresponding expression of genes involved in the process in both cardiac tissue and blood from the rat subjected to I/R injury.

## Materials and methods

### Animals

All experiments involving the animals were reviewed and approved by Institutional Animal Ethics Committee (IAEC), SASTRA Deemed University, Thanjavur, India (CPCSEA Approval No. 650/SASTRA/IAEC/RPP) and were conducted following the CPCSEA (Committee for the Purpose of Control and Supervision of Experiments on Animals) guidelines. Around 24 male Wistar rats (200–250 g) of 8–12 weeks old were used for this study from the Central Animal Facility at SASTRA Deemed University, Thanjavur, India. Animals were housed in polycarbonate cages and maintained at 25 ± 2°C with 12 h light/dark cycle and relative humidity of 65 ± 2%. Feed and water were provided *ad libitum*.

### Left anterior descending artery ligation model and experimental groups

Male Wistar rats were randomly divided into four groups (*n* = 6/group). (1) Normal (N), (2) I/R control, (3) DNMT inhibition control (Di-C), and (4) DNMT inhibition I/R (Di-I/R). The rats were anesthetized with halothane (1.5% O_2_) and placed on a thermal heating pad to maintain 37°C. The rats were tested for the loss of pedal reflex post-anesthesia, so the animal experienced no pain or stress during surgery. The rats were supported on a rodent ventilator (70 strokes/min at a tidal volume of 10 mL/kg). I/R operated rats underwent a 10 min stabilization time followed by an incision between the 3rd and 4th intercostal ribs. The pericardium of the heart was detached, and a 7–0 suture was passed below the left anterior descending artery (LAD). A slipknot was made to occlude the blood flow to create ischemia for 30 min, and the knot was removed to create reperfusion to the heart for 1 h. The normal rats underwent the same procedure without LAD occlusion. Di-C and Di-I/R group rats were pre-treated with the DNA methyltransferase (DNMT) inhibitor 5-azacytidine in phosphate-buffered saline (PBS) 5 mg/kg intraperitoneally for 15 alternate days, where the animals did not receive any drug on 16th day (8). On the 17th day, along with the normal and I/R groups, the Di-C and Di-I/R group rats were subjected to normal group and I/R group protocol, respectively. The appearance of regional epicardial cyanosis confirmed ischemia, and reperfusion was confirmed with the visualization of the arterial blood flow.

### Sample collection and processing

The peripheral blood (5 mL) was drawn from the abdominal aorta. Immediately after collection, the blood was processed to separate peripheral blood mononuclear cells (PBMCs) and plasma. Briefly, the blood was diluted with PBS (1:4) and layered onto density gradient separation media (HiSep™-LSM-1077, Himedia, Thane, India). The tubes were centrifuged at 2,000 rpm for 20 min at 10°C without brakes to separate the PBMC from RBC and plasma. The PBMC fraction was collected and washed with PBS and stored at −80°C for further analysis. The heart was collected and flash frozen in liquid nitrogen and stored at −80°C. The left ventricle portion of the heart was further utilized for analysis.

#### Isolated rat heart model and experimental groups

Eight to twelve weeks old male Wistar rats (200–250 g) were divided into four groups on a random basis (*n* = 6/group): (1) Normal (N), (2) I/R, (3) DNMT1i control (Di-C), (4) DNMT1i I/R (Di-I/R). The rats were anesthetized with sodium thiopentone (60 mg/kg1 i.p.). The hearts were excised, mounted on a Langendorff apparatus, and perfused with Krebs–Henseleit (KH) buffer per the treatment groups.

The rats in the normal groups (N) were perfused continuously with KH buffer for 120 min. In contrast, the I/R group rat heart (I/R) underwent 20 min of stabilization with KH buffer perfusion, followed by ischemia for 30 min (Buffer flow was stopped) and reperfusion for 60 min (restoring the KH buffer flow to the heart).

The Di-C and Di-I/R groups were pre-treated with 5-azacytidine (5 mg/Kg b.wt., intraperitoneally) in PBS every alternate day for 15 days, followed by perfusion as per normal and I/R group, respectively.

### Ischemia reperfusion injury assessment by hemodynamics evaluation (isolated rat heart model)

Cardiac recovery was assessed by monitoring the hemodynamic changes in the heart’s left ventricle by inserting a pre-loaded balloon into the left ventricle (left ventricular pressure). LabChart Pro 8 and Power Lab Data Acquisition System (AD Instruments, New South Wales, Australia) were used to record the left ventricular pressure changes. The heart rate (HR), left ventricular developed pressure (LVDP), and left ventricular end diastolic pressure (LVEDP) were calculated from the left ventricular pressure values.

### Histopathological examination

The heart tissues were fixed in 10% formalin and were made into tissue blocks after embedding in paraffin. The blocks were then cut into 5-μm sections and stained with hematoxylin and eosin (H&E). The sections were then examined under a light microscope (Nikon, Tokyo, Japan) for the pathological changes at 40× magnification.

### Infarct size measurement

The hearts were fixed with 4% formalin solution for 24 h post-experiment. The transverse tissue sections of the heart were stained with 1.5% 2,3,5-triphenyl tetrazolium chloride (TTC) for 30 min at 37°C. They were quantified for the extent of myocardial tissue damage using ImageJ analysis software (NIH, Maryland, USA) (14).

### Cardiac injury markers estimation

Myocardial injury was evaluated by measuring the activity of lactate dehydrogenase (LDH) and creatine kinase (CK-MB) enzymes in both plasma and tissue following the standard protocol described by Sigma-Aldrich, Missouri, United States (MAK066) and Agappe, Kochi, India (11405007) kit. Myeloperoxidase (MPO) activity was assessed as an inflammatory biomarker in PBMC, according to the protocols mentioned elsewhere (15). mRNA expression of the injury marker dysferlin was assessed in both blood and tissue RNA using real-time PCR analysis. The primer sequence of the gene is given in Table 1.

**TABLE 1:** Primer sequence details: The forward and reverse primer sequences of the genes used for real-time PCR analysis are presented.

S.No.	Gene	Forward primer	Reverse primer
1	GAPDH	5′-GCGAGATCCCGCTAACATCA-3′	5′-CTCGTGGTTCACACCCATCA-3′
2	DNMT1	5′-CGGATTGTCGGATAAAAGA-3′	5′-GCTTCCTCATCGCTCCAGTA-3′
3	DNMT 3A	5′-GGAGAGGAAAGGGAGAGAGG-3′	5′-AGGGATGGTGCTGGTGAGAC-3′
4	DNMT3B	5′-AAACCCAACAACAAGCAACC-3′	5′-ACATCAGAAGCCATCCGTTC-3′
5	TET1	5′-TATATGGCTGTGCTGTGCTGCCCAA-3′	5′-CGATGGGCCATTGCTTGATG-3′
6	TET2	5′-TGTTGTCAGGGTGAGAATCCAG-3′	5′-CCTGTAGGCATCAGGTGCAA-3′
7	TET3	5′-CCCTTGCCTGAAGCATCTCA-3′	5′-GCCGAGGTACCATTCCCAAA-3′
8	Casp9	5′-GAGGATATTCAGCGGGCAGG-3′	5′-GCAGGAGATGAAGCGAGGAA-3′
9	Casp3	5′-CGGACCTGTGGACCTGAAAA-3′	5′-TAACCGGGTGCGGTAGAGTA-3′
10	Casp7	5′-TTCGACGGAAGACGGAGTTG-3′	5′-CCGGACATCCATACCTGTCG-3′
11	PARP	5′-ACCACGCACAATGCCTATGA-3′	5′-AGCAGTCTCCGGTTGTGAAG-3′
12	DYSF	5′-AAGAGGAGCCTGCAGGTGTA-3′	5′-TGTGTTGAGCTCCGCATAAG-3′
13	IL1B	5′-CGACAAAATCCCTGTGGCCT-3′	5′-GGGTGTGCCGTCTTTCATCA-3′
14	TLR4	5′-GAGAAGTCCTTGCTGAGGCA-3′	5′-TCCCACTCGAGGTAGGTGTT-3′
15	ICAM1	5′-AGGTATCCATCCATCCCACA-3′	5′-GCCACAGTTCTCAAAGCACA-3′
16	MyD88	5′-GAGCAGTGTCCCACAGACAA-3′	5′-AGTAGCAGATGAAGGCGTCG-3′
17	POLG	5′-CTTTGGGCTCCAGCTTGACT-3′	5′-TGGAGAAAATGCTTGGCACG-3′
18	PGC 1α	5′-GAGGGACGAATACCGCAGAG-3′	5′-CTCTCAGTTCTGTCCGCGTT-3′
19	TFAM	5′-GTTGCTGTCGCTTGTGAGTG-3′	5′-GTCTTTGAGTCCCCCATCCC-3′
20	ND1	5′-CCACCGCGGTCATACGATTA-3′	5′-AGGGCTAAGCATAGTGGGGT-3′
21	CYTB	5′-ACAAAATCCCATTCCATCCA-3′	5′-GTTGGGAATGGAGCGTAGAA-3′
22	ND6	5′-ATCCGGAAACTTGAGGGTCT-3′	5′-CCCAGCCACCACTATCATTC-3′
23	ND5	5′-ATTGCAGCCACAGGAAAATC-3′	5′-TGGTGATTGCACCAAGACAT-3′
24	ND4L	5′-GGTACTTTTATATTTCGCTCCCACT-3′	5′-CGCAGGCTGCAAAAACTAGA-3′
25	ND3	5′-TGCATTCTGATTGCCTCAAA-3′	5′-TGGGAGGGGGAGTAGTAAGG-3′
26	COX3	5′-AGCCCATGACCACTAACAGG-3′	5′-TGGCCTTGGTATGTTCCTTC-3′
27	ATP6	5′-ACACCAAAAGGACGAACCTG-3′	5′-AGAATTACGGCTCCTGCTCA-3′
28	ATP8	5′-ACACCAAAAGGACGAACCTG-3′	5′-AGAATTACGGCTCCTGCTCA-3′
29	COX2	5′-GCTTACAAGACGCCACATCA-3′	5′-GAATTCGTAGGGAGGGAAGG-3′
30	COX1	5′-AATTGGAGGCTTCGGAAACT-3′	5′-CTGTTCCAGCTCCAGCTTCT-3′
31	ND2	5′-AAAAAGCCCACGATCAACTG-3′	5′-GGGAATTCCTTGGGTGACTT-3′
32	ND4	5′-CCCACTCTTAATTGCCCTCA-3′	5′-CGTGGGCTTTTGGTAATCAT-3′
33	SOD1	5′-CGGATGAAGAGAGGCATGTT-3′	5′-CAATCACACCACAAGCCAAG-3′
34	Catalase	5′-ACCAAGGTTTGGCCTCACAA-3′	5′-GAGCACGGTAGGGACAGTTC-3′
35	Gpx1	5′-CCGATATAGAAGCCCTGCTG-3′	5′-GAAACCGCCTTTCTTTAGGC-3′
36	NFKB	5′-CTGTCCTCTCGCATCCGATT-3′	5′-AGTTCCGGTTTACTCGGCAG-3′

### Gene expression analysis

RNA was isolated from blood and cardiac tissue samples using TRIzol reagent and further converted into cDNA using the cDNA synthesis kit protocol (Thermo Fisher Scientific, Waltham, MA, USA). The mRNA expression of the samples was assessed using DyNAmo Flash SYBR green (Thermo Fisher Scientific) via the real-time PCR analysis for the genes involved in methylation (DNMT1, DNMT 3A, DNMT3B, TET1, TET2, and TET3); injury (Dysf); apoptosis (CASP3, CASP7, CASP9, and PARP); Oxidative stress [CuZnSOD, Catalase, NFKB, glutathione peroxidase (Gpx)]; mitochondrial electron transport chain (ETC) function (Mitochondrial encoded complex I genes: ND1, ND2, ND3, ND4, ND4L, ND5, and ND6; Complex III genes: Cyt B; Complex IV genes: COX1, COX2, and COX3; Complex V genes: ATP6 and ATP8); mitochondrial replication genes (PGC-1α, POLG, and TFAM); apoptosis (Casp9, Casp3, Casp7, and PARP); and inflammation (IL-1β, TLR4, ICAM1, and MyD88) on the system ABI 7500 (Applied Biosystems, Foster City, CA, USA) The primer sequence of the genes are given in Table 1. The relative gene expression was calculated by the method of Livak and the expression of the genes was normalized with the GAPDH gene (16).

### DNA methyltransferase enzyme activity

The DNMT enzyme activity was evaluated using the EpiQuik™ DNA methyltransferase Activity/Inhibition Assay Ultra Kit in the nuclear extract from blood PBMC and tissue. The fluorescence values, which are formed as a result of the interaction of methylated DNA with anti-5-methylcytosine antibody, were measured to calculate the activity.

### Global DNA methylation analysis

DNA was isolated from the rat heart (left ventricular region) and blood samples using Phenol/Chloroform/Isoamyl alcohol method (17). Mitochondrial DNA (mtDNA) was isolated from the cardiac tissue (left ventricular region) as per the protocol mentioned by Isakallio et al. (18). Briefly, mitochondria were isolated from the cardiac tissues (19), followed by DNase I and RNase A treatment to remove the nuclear DNA and RNA, respectively. Mitochondrial DNA was then isolated by chloroform: isoamyl alcohol and further precipitated with ethanol. The absence of contamination in mtDNA samples with the nuclear DNA was confirmed by the absence of expression of the nuclear-encoded GAPDH gene in mtDNA. Global DNA methylation was analyzed in nuclear and mtDNA using MethylFlash™ Global DNA Methylation (5-mC) ELISA Easy Kit (Epigentek, Newyork, United States).

### Apoptosis detection

The apoptotic rate of cells in myocardial tissue was evaluated according to the manufacturer’s instructions using TdT-mediated biotinylated nick end labeling (TUNEL) Assay Kit (MK500, Takara biosciences, New Delhi, India). The sections were then analyzed under a fluorescence microscope in 20× magnification and the number of TUNEL-positive cells in each visual field was evaluated. Apoptosis was further confirmed by estimating the levels of the apoptotic executioner, the Caspase-3 enzyme using the method of Gilbert et al. (20).

### Mitochondrial DNA copy number estimation

Mitochondrial DNA copy number was calculated as the ratio of relative gene expression of MT-ND1 gene and GAPDH gene expression using DNA as a source sample (21).

### Mitochondrial isolation and evaluation of mitochondrial functional activities

Mitochondria were isolated by density-gradient centrifugation using the homogenized heart tissue as per the protocol mentioned in Palmer et al. (19). Briefly, the tissue homogenate, prepared in isolation buffer (100 mM KCl, 40 mM Tris HCl: pH 7.5, 10 mM tris base, 40 mM MgCl_2_, 1 mM EDTA, 1 mM ATP) was centrifuged at 4°C, 600 × *g* for 10 min. The resultant supernatant was re-centrifuged at 6,000 × *g* (4°C) for 10 min to yield the mitochondrial pellet. The pellet was then dissolved in an isolation buffer containing 0.1% BSA and centrifuged for 10 min at 4°C, 12,000 × *g* for 10 min. The resulting mitochondrial pellet was dissolved in a storage buffer.

Mitochondrial ATP content was estimated in the samples using the ATP lite (Perkin Elmer, Waltham, United States) kit as per the manufacturer’s instructions. Further, mitochondrial ETC activities were assessed in the mitochondria post-disruption of the mitochondrial membranes by freeze-thawing (3×) in a hypotonic medium (25 mM K_2_HPO_4_ pH 7.2, 5 mM MgCl_2_). The activities of the complex I (NQR), complex II (SQR), complex III (QCR), and complex IV (COX) were measured spectrophotometrically by using the complex specific acceptors and donors, as described elsewhere (22).

### Oxidative stress analysis

The total reactive oxygen species (ROS) level in the cardiac tissue was analyzed by measuring the fluorescence at Ex/Em = 485/530 nm using 2′,7′-dichlorofluorescein diacetate (DCHFDA) (Cat No. D6883, Sigma Aldrich, Missouri, United States). The redox status of glutathione was assessed by measuring the level of reduced glutathione (GSH) and oxidized glutathione (GSSG) in the heart using the method of Shaik et al. (23). The activities of antioxidant enzymes Gpx, catalase and superoxide dismutase (SOD) were estimated using the standard methods described elsewhere (24, 25).

### Statistical analysis

All data were represented as the mean ± SD. The significance level between the groups was assessed with a one-way ANOVA test followed by Dunnett’s test, a *post hoc* analysis using Graph Pad Prism 7.0 software. Correlation analysis was performed using the Pearson coefficient method.

## Results

### Ischemia reperfusion induced alterations in global DNA methylation and gene expression of its associated enzymes in the myocardium and blood: Relation with cardiac injury

**FIGURE 1:**
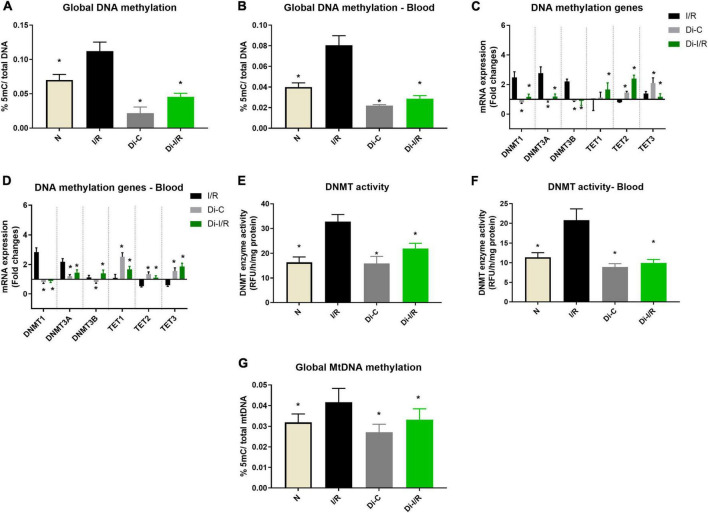
Ischemia reperfusion (I/R) induced DNA methylation changes in the myocardium and blood in LAD model. Methylation changes induced by I/R were assessed from **(A)** %5-mC in cardiac DNA; **(B)** %5-mC in blood DNA; **(C)** DNMT and TET gene expression in the myocardium; **(D)** Blood DNMT and TET gene expression; **(E)** Myocardial DNMT activity; **(F)** DNMT activity in blood; **(G)** mtDNA methylation in the myocardium. The graphs represent mean ± SD values. The changes in gene expression are represented as fold changes from the normal group. *p < 0.05 vs. I/R.

Reperfusion of the ischemic heart induced an elevated global DNA methylation by 47% in the myocardium from the normal heart (Figure 1A). In the blood compartment, the global DNA methylation level was increased in PBMC by 50% upon I/R injury from the normal group (Figure 1B). Further analysis on the mRNA expression level of the methylating and demethylating enzymes (DNMT and TET, respectively) in the myocardium (that contribute to the global DNA methylation changes), showed that I/R rat exhibited a significant increase in DNMT1, 3A and 3B gene expression of the myocardium by 2.4, 2.7, and 2.2 folds, respectively (Figure 1C) from the normal group. On the other hand, the expression of the demethylating enzymes TETs did not exhibit any significant changes in the I/R myocardium from the normal heart. In the blood compartment, I/R upregulated only DNMT1 (2.1 folds) and 3A (2.9 folds) from the normal group (Figure 1D). Besides, the blood from I/R rats showed significant downregulation of TET2 and TET3 by 0.53 and 0.59 folds, respectively unlike the insignificant TET changes in the myocardium. The enzyme activity of DNMT measured in the I/R myocardium and blood exhibited 50 and 52% elevation, respectively (Figures 1E,F) from normal heart, which is also reflected in the increased global DNA methylation level (Figures 1A,B).

Further analysis on global mtDNA methylation showed that I/R imparted a global DNA hypermethylation of mtDNA by 34% in hearts. Inhibition of DNMT not only reduced the nuclear DNA methylation by 54% from I/R but also reduced the mtDNA methylation by 21% when compared with the I/R hearts (Figure 1G).

**FIGURE 2:**
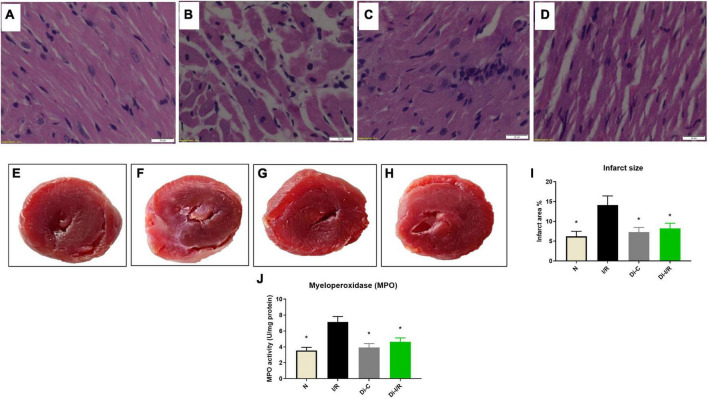
Targeting DNA methylation via its inhibitor improved the I/R-altered myocardial histology and reduced the infarct size; and blood MPO levels. Histopathological examination of myocardium in LAD model from rat hearts of group **(A)** normal; **(B)** I/R; **(C)** Di-C; **(D)** Di-I/R. The representative images were obtained at 40× magnification and the scale bars indicate 20 μM. The representative TTC stained images subjected to the perfusion protocol as per the groups: **(E)** Normal; **(F)** I/R; **(G)** Di-C; **(H)** Di-I/R; **(I)** represents the percentage of infarct size; **(J)** represents MPO activity in blood. The graph represents the mean ± SD of the percentage of the area of infarct size. *p < 0.05 vs. I/R.

**FIGURE 3:**
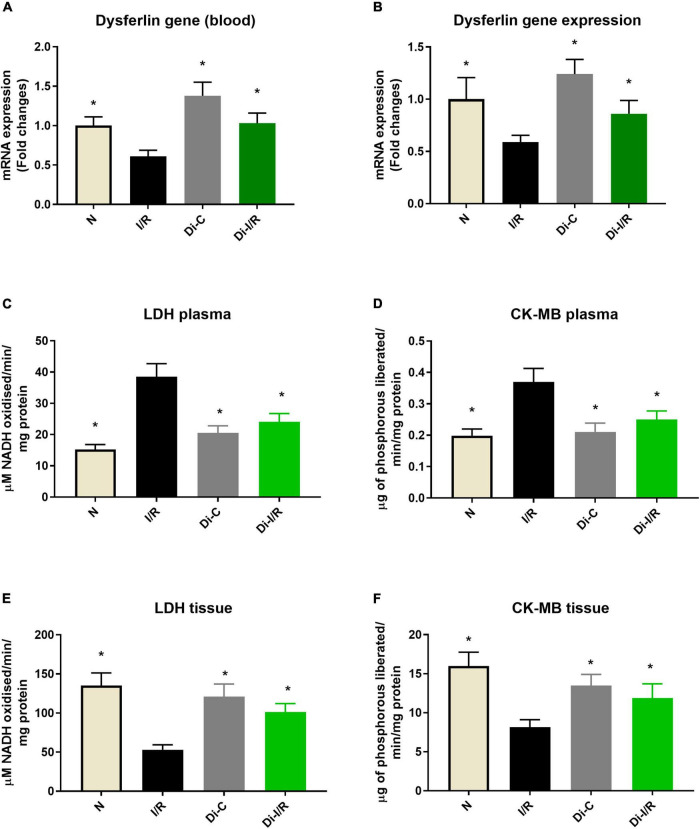
DNA methylation inhibitor pre-treatment altered the I/R-associated cardiac injury markers in the heart and blood. **(A)** The dysferlin mRNA expression levels in the blood; panel **(B)** represents the dysferlin mRNA expression levels in the heart. The cardiac injury markers lactate dehydrogenase and creatine kinase were evaluated from the **(C,D)** plasma and **(E,F)** myocardium, respectively. The changes in gene expression are represented as fold changes from the normal group. The graphs represent mean ± SD values. *p < 0.05 vs. IR.

Further, the I/R-associated injury was assessed in the myocardium and blood, as shown in Figure 2A, I/R challenged rat hearts exhibited alterations in cellular architecture with higher inflammatory cell infiltration, interstitial edema, and a few myocardial fibers broken, indicating injury. The tissue injury was further analyzed with TTC staining, where the rat hearts subjected to I/R exhibited an increased infarct size by 47% (Figure 2I) from the normal heart. On the other hand, the blood inflammatory marker MPO activity in leukocytes was found to be increased in I/R rats by 52% in I/R blood from the normal group (Figure 2J). Furthermore, blood analysis showed that I/R imparted a significant decline in cardiac injury marker dysferlin gene expression by 41% in the blood (Figure 3A), in coherence with the 39% decline of dysferlin gene expression in the myocardium (Figure 3B). Moreover, the cardiac injury markers LDH and CK-MB were found to be significantly elevated in the plasma of I/R rats by 54% and 48%, respectively (Figures 3C,E) with corresponding lower levels in the myocardial tissue when compared with normal hearts (Figures 3D,F), indicating the cardiac I/R injury reflection in blood.

Targeting the DNA methylation by inhibiting the DNMT in rats prior to I/R induction with 5-azacytidine (Di-I/R) showed a significant reduction in the global DNA methylation levels by 54% in the myocardium (Figure 1A). Blood DNA methylation analysis of Di-I/R rats showed that global DNA methylation in blood was significantly reduced by 38% in Di-I/R rats when compared with I/R (Figure 1B). In the myocardium, DNMT inhibition reversed the expression of DNMT1, 3A, 3B, TET1, TET3 genes to a near-normal level. But, TET2 gene expression showed an upregulation by 3.1 folds in DNMTi_I/R myocardium when compared with the I/R group (Figure 1C). Similar in the blood, DNMT inhibition brought the expression of DNMT1, DNMT3A, DNMT3B, TET3 genes to normal levels, along with an upregulation in TET1 and TET2 gene expression by 2 and 2.4 folds, respectively (Figure 1D). In fact, I/R-associated elevated DNMT activity was reduced to 38% in the myocardium and by 33% in blood in Di-I/R rats (Figures 1E,F).

The relationship of DNA methylation with I/R injury was assessed further by measuring the injury in the myocardium and blood of Di-I/R rats. Di-I/R rat hearts reduced I/R-induced myocardial tissue lesions and maintained intact myocardial architecture (Figure 2D). The reduced pathological changes in the Di-I/R heart were reconfirmed with TTC staining, where DNMT inhibition in Di-I/R rats reduced the infarct size by 44% and increased the dysferlin gene expression by 34% in the myocardium from I/R group, confirming the direct relation of DNA methylation with myocardial injury (Figures 2I, 3A). On the other hand, the DNMTi treatment reduced the blood MPO activity by 36% from the I/R (Figure 2J) and improved the dysferlin mRNA expression in blood to near-normal levels in rats (Figure 3B), emphasizing the key role played by methylation in blood as well. Moreover, the cardiac injury markers LDH and CK-MB, were reduced in the plasma of Di-I/R rats by 44% and 36% respectively (Figures 3D,F) from the I/R group with a corresponding improvement in their levels in the myocardium (Figures 3C,E), reconfirming the significant association of DNA methylation with I/R injury.

### Isolated rat heart model reveals the influence of blood in ischemia reperfusion-associated DNA methylation in the heart

**FIGURE 4:**
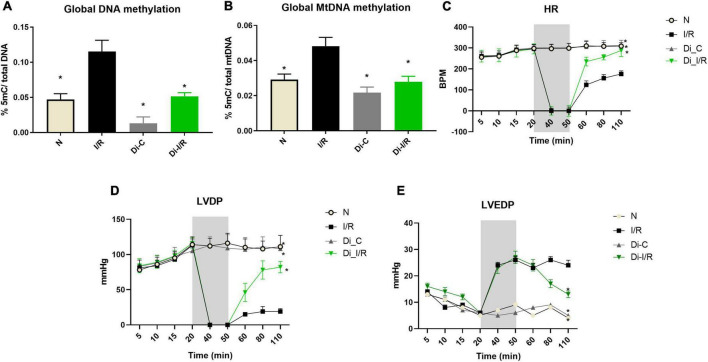
Ischemia reperfusion (I/R) induced DNA methylation changes in the isolated rat heart model and compromised the cardiac function. Methylation changes induced by I/R was assessed in hearts of isolated rat heart from **(A)** %5-mC in nuclear DNA; **(B)** %5-mC in mitochondrial DNA. The changes in the hemodynamic indices were evaluated from **(C)** heart rate (HR); **(D)** LVDP; **(E)** LVEDP. *p < 0.05 vs. I/R.

The influence of blood-borne components in modulated DNA methylation in the heart was assessed by using an isolated rat heart perfusion model (absence of blood) and the results are given in Figure 4. Accordingly, I/R-induced DNA hypermethylation by 56% from the normal heart than the observed value in LAD model (47% in I/R hearts). DNA methylation inhibition reduced the global DNA methylation level by 45% from I/R hearts (Figure 4A). We further assessed the mitochondrial genome methylation level and the results showed that I/R exhibited a 41% increase in global mtDNA methylation from normal hearts (Figure 4B). DNMT inhibition reduced global mtDNA hypermethylation by 33% from I/R hearts (Figure 4B) in the absence of blood.

Further analysis of the hemodynamic data showed that I/R imparted a significant decline in the HR, LVDP, and elevated LVEDP from normal hearts. Inhibition of DNMT prior to I/R preserved the cardiac hemodynamics, evident by the improvement in cardiac LVDP by 74% in Di-I/R hearts when compared with the I/R hearts (Figures 4C–E).

### DNA methylation regulates the apoptosis and inflammation in the myocardium and blood during ischemia reperfusion

**FIGURE 5:**
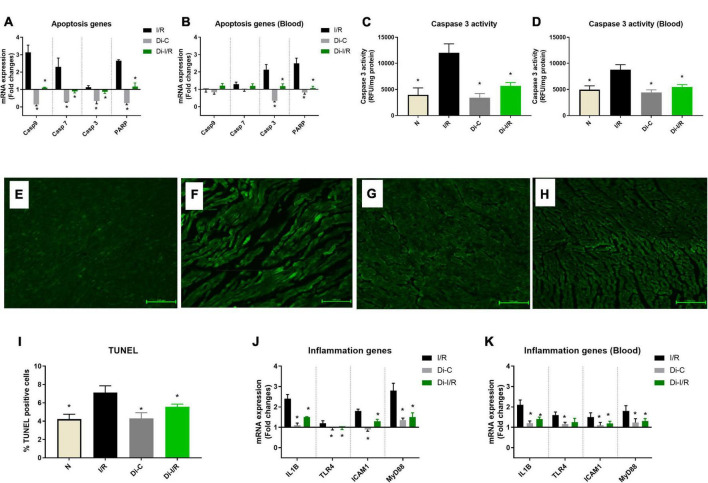
Targeting DNA methylation during I/R reduced the apoptosis and inflammation in the myocardium and blood. The methylation impact of I/R on apoptosis and inflammation was assessed from **(A)** mRNA expression changes in the apoptosis genes in the heart; **(B)** Blood mRNA expression changes in the apoptosis genes; **(C)** Myocardial Caspase 3 activity; **(D)** Blood caspase 3 activity; The representative TUNEL stained images of the groups **(E)** normal; **(F)** I/R; **(G)** Di-C; **(H)** Di-I/R were obtained at 20× magnification and the scale bars indicate 100 μM. **(I)** Represents the apoptotic positive cells (TUNEL) percentage. Panel **(J)** represents the mRNA expression changes in inflammation genes in the myocardium and **(K)** represents the mRNA expression changes of inflammation genes in the blood. The graph represents the mean ± SD. The changes in gene expression are represented as fold changes from the normal group. *p < 0.05 vs. I/R.

Apoptosis and inflammation are one of the key pathological features of I/R injury. I/R induction resulted in a significant upregulation in the apoptotic genes, including caspase 3,7 and PARP, by 3.1, 2.2, and 2.6 folds, respectively, in the myocardium. In contrast, caspase 9 did not show any significant variation (Figure 5A). Similarly, blood apoptotic gene expression analysis in I/R rats displayed a significant upregulation in the apoptotic genes, caspase 3,7 and PARP by 2.1, 1.3, and 2.4 folds, respectively in the blood without much variation in caspase 9 expression, similar to the myocardial tissue (Figure 5B). A corresponding elevation in caspase 3 activity (43%) in blood was observed in I/R rat (Figure 5D). TUNEL staining was performed to detect DNA breakage in final stage of apoptosis (Figures 5E–H). The count of TUNEL-positive cells was higher in the I/R rat heart (33%) (Figure 5I).

Figure 5J shows the differential inflammatory gene expression pattern of I/R rat hearts compared to the normal group. I/R upregulated the mRNA expression of IL-1β, TLR4, ICAM1, and MyD88 by 2.4, 1.2, 1.8, and 2.8 folds, respectively, in the hearts (Figure 5J), with a similar pattern of upregulation in blood by 2.6, 1.6, 1.5, and 1.8 folds, respectively (Figure 5K).

Inhibition of DNMT reversed the I/R-associated changes in apoptotic caspases mRNA expression to near-normal level (Figures 5A,B) in both myocardium and blood of Di-I/R rats, which was reflected in a significant reduction in caspase 3 enzyme activity by 39% in blood and 61% in the myocardium (Figures 5C,D), when compared with I/R rats. Moreover, the inhibition of DNMT reduced the extent of apoptotic cell death in hearts (measured via TUNEL) by 28% from the I/R group (Figure 5I).

Further analysis of inflammatory genes showed that inhibition of DNMT1 prior to I/R reduced the gene expression in IL-1β, TLR4, ICAM1, and MyD88 genes to 1.5, 0.98, 1.3, and 1.5 folds, respectively, from the normal control heart (Figure 5J). Although DNMT inhibited I/R rats did not alter the inflammatory gene expression in blood to near-normal level, the expression was reduced from I/R significantly by 33%, 21%, 20%, and 27% respectively, when compared with I/R hearts (Figure 5K).

### DNA methylation regulates the mitochondrial function in the myocardium during ischemia reperfusion

**FIGURE 6:**
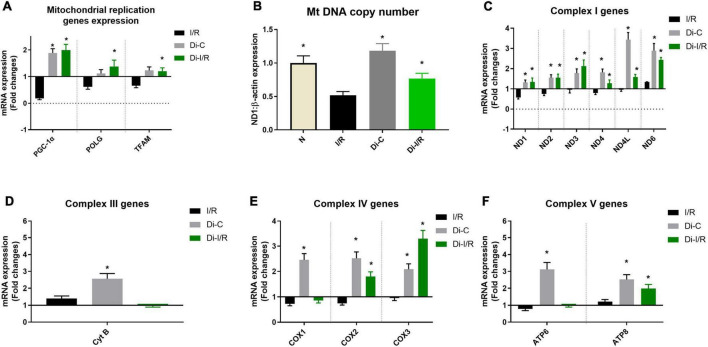
DNA methyltransferase (DNMT) inhibitor pre-treatment upregulated the mitochondrial replication and mitochondrial encoded ETC genes and improved the mitochondrial copy number during I/R. **(A)** The mRNA expression changes of replication control genes; Panel **(B)** represents the mitochondrial DNA copy number; The mRNA expression changes of mitochondrial encoded ETC genes were presented for **(C)** Complex I; **(D)** Complex III; **(E)** Complex IV; **(F)** Complex V. The graph represents the mean ± SD. The changes in gene expression are represented as fold changes from the normal group. *p < 0.05 vs. I/R.

Impaired mitochondrial biogenesis and mitochondrial oxidative phosphorylation play a pivotal role in the pathology of I/R. Figure 6A shows differential expression of mitochondrial biogenesis, replication and transcription control genes in I/R and Di-I/R hearts compared to the normal rat heart. The master regulator of mitochondrial biogenesis, PGC-1α, the mitochondrial polymerase, POLG, and the mitochondrial transcription factor TFAM were significantly downregulated to 0.18, 0.62, and 0.66 folds, respectively, from normal in I/R rat hearts (Figure 6A). Targeting the DNA methylation with DNMTi prior to I/R upregulated the biogenesis genes PGC-1α, POLG, and TFAM to 1.99, 1.38, and 1.20 folds, respectively from normal. The improvement in the biogenesis gene expression upon targeting DNA methylation was reflected in the corresponding improvement in mtDNA copy no., by 22% in Di-I/R rat hearts compared with I/R hearts. I/R hearts exhibited a 36% decline in mitochondrial copy number from normal group rat hearts (Figure 6B).

Further analysis of the expression of genes involved in mitochondrial OXPHOS function encoded by mtDNA showed the downregulation of nine genes out of 13 genes in the I/R rat heart. However, with I/R only six genes ND1 (0.57 folds), ND2 (0.57 folds), ND4 (0.79 folds), COX1 (0.73 folds), COX2 (0.75 folds), ATP6 (0.78 folds) were significantly downregulated from normal hearts (Figures 6C–F). Targeting the DNA methylation in Di-I/R hearts upregulated all the 13 genes above normal level except the COX1 gene (downregulation by 0.86 folds from normal).

**FIGURE 7:**
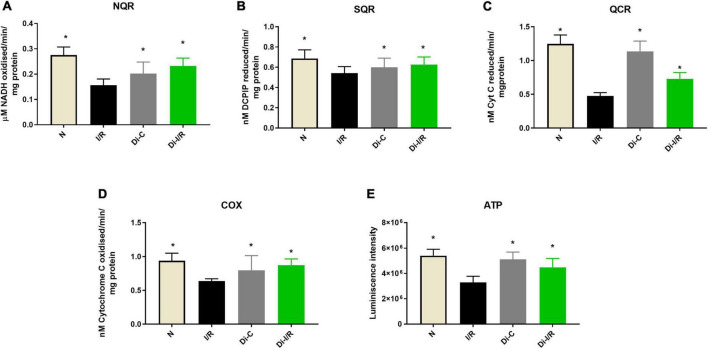
Inhibition of DNA methylation improved the mitochondrial function post-I/R. The effect of DNA methylation inhibition on mitochondrial function was assessed by evaluating the electron transport chain enzyme activities of complexes **(A)** NQR (Complex I); **(B)** SQR (Complex II); **(C)** QCR (Complex III); **(D)** COX (Complex IV). Panel **(E)** shows the ATP content. The graphs represent mean ± SD values. *p < 0.05 vs. I/R.

The end effector of the changes in the mtDNA gene expression was verified in the mitochondria by assessing the bioenergetics function. Accordingly, I/R rat heart exhibited declined complex I, II, III, and IV activities by 54%, 28%, 62%, and 43% respectively, with a significantly declined ATP level by 39% when compared with normal hearts (Figures 7A–D). Inhibiting the global DNA methylation in hearts prior to I/R improved the complex activities by 46%, 18%, 51%, and 39%, respectively in Di-I/R hearts, with a corresponding improvement in ATP levels by 35% when compared with I/R hearts (Figure 7E).

These observations suggest that targeting the global DNA methylation in I/R hearts could recover mitochondrial biogenesis by regulating the genes PGC-1α, POLG, and TFAM and the bioenergetics functions via upregulating the mtDNA encoded 13 bioenergetic genes except COX1.

### DNA methylation regulates the oxidative stress in the myocardium during ischemia reperfusion

**FIGURE 8:**
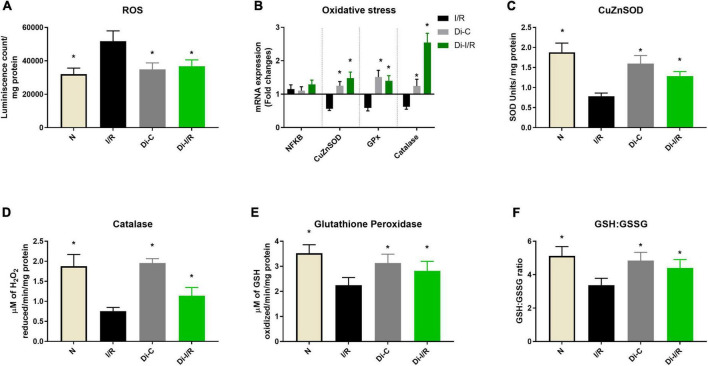
mRNA expression and activity changes of oxidative stress response during DNA methylation inhibition. Panel **(A)** represents the ROS levels; **(B)** mRNA expression changes in antioxidant response genes (represented as fold changes from the normal group); Enzyme activities of antioxidant enzymes **(C)** superoxide dismutase (SOD); **(D)** catalase; **(E)** glutathione peroxidase; **(F)** GSH: GSSG ratio. *p < 0.05 vs. I/R.

The dysregulation of the mitochondrial OXPHOS often results in the burst of ROS, accumulating oxidative stress. Accordingly, our results showed that I/R increased the ROS levels in the heart by 39% from normal, and Di-I/R rat hearts reduced the ROS levels by 27% from I/R hearts (Figure 8A).

Further, we analyzed the mRNA expression of the antioxidant genes in I/R and Di-I/R hearts to understand the impact of DNA methylation on the expression of antioxidant enzyme genes. I/R imparted a significant downregulation in the mRNA expression of the antioxidant enzymes such as SOD1, Gpx2, and catalase by 0.56, 0.59, and 0.62 folds, respectively, from normal without much change in the expression of NFκB gene (Figure 8B). The changes in the gene expression was reflected in their activities as well, where the I/R significantly decreased the SOD, catalase and GPx enzyme activity by 59%, 48%, and 37%, respectively (Figures 8C–E) and decreased the GSH/GSSG ratio by 34% (Figure 8F).

Targeting the DNA methylation with DNMT inhibitor before I/R insult could significantly upregulate the I/R-declined SOD1, Gpx2, and catalase genes by 1.48, 1.40, and 2.54 folds, respectively, from the normal group (Figure 8B). This was in coherence with the improvement in the activities of the corresponding antioxidant enzymes, where SOD, catalase and GPx activity were improved by 45%, 38%, and 26%, respectively (Figures 8C–E) and elevated the GSH/GSSG ratio by 28% (Figure 8F).

### Correlation analysis between blood and cardiac DNA methylation: Impact on injury

Pearson’s correlation analysis was performed to understand the relationship between DNA methylation in blood and tissue with different pathological parameters linked to I/R pathology.

The association of the cardiac global DNA hypermethylation with the cardiac injury parameters (LDH, CK-MB, Dysferlin, infarct size) in I/R hearts was assessed via correlation analysis. Our results showed a significant negative correlation of the global DNA methylation level only with infarct size (*r* = −0.7011, *p* = 0.0489).

Meanwhile, Pearsons coefficient analysis of blood global DNA methylation with the cardiac injury markers in blood (LDH, CK-MB, dysferlin, MPO) showed a significant negative correlation between global DNA methylation with blood dysferlin mRNA expression values (*r*-value = −0.6741, *p*-value = 0.0429).

Once we found that the blood and tissue global DNA methylation negatively correlated with their corresponding cardiac injury markers in their respective compartments during I/R injury, as a next step forward, we further checked if the blood methylation level can be a predicting factor for assessing the cardiac I/R injury in the heart via correlation analysis.

The correlation data showed that global blood DNA methylation level did not correlate with infarct size, but it showed a significant negative correlation with myocardial dysferlin levels (*r* = −0.7602, *p* = 0.0482).

## Discussion

Despite the promising scientific advancements, successful therapy or approach in preventing I/R remains elusive. The present therapeutic targets that focus on blocking or scavenging free radicals and preventing cell death by targeting pro-death pathways and activating pro-survival pathways did not fetch satisfactory results in clinical settings, despite preclinical success (26). Based on this knowledge, the search for new therapeutic targets persists, where we focus on the upstream regulator of I/R pathological mediators via controlling the gene expression. In this direction, we explored the therapeutic potential of DNA methylation that controls the battery of genes involved in the preclinical successful targets and strategies. The major findings of the present study are as follows. (1) Reperfusion of ischemic hearts induced an elevation of global DNA hypermethylation and this hike in methylation was reversed with inhibition of DNMT1, the enzyme responsible for the DNA methylation. Importantly, Pearsons’ correlation analysis showed a positive relationship between infarct size and DNA hypermethylation, which was experimentally proved in the present study by measuring the infarct size of I/R hearts in the presence and absence of DNMT inhibitor (to block DNA methylation). (2) The impact of DNA methylation on the I/R heart was then evaluated with respect to cellular mediators like oxidative stress and mitochondria, where we noted a significant reduction in I/R-associated oxidative stress and improvement in mitochondrial function in I/R rat heart treated with DNMT inhibitor. (3) Further, blood analysis showed a significant elevation in DNA hypermethylation in PBMC of I/R rat blood and was decreased prominently in DNMT inhibitor treated I/R rat blood. In fact, circulatory cardiac injury markers like CK-MB and LDH were high with significant downregulation of dysferlin gene expression. Importantly, elevated DNA methylation in the blood showed a negative correlation with the blood dysferlin gene expression. (4) To assess whether the cardiac DNA hypermethylation induction was influenced by the blood-borne mediators derived during I/R, we performed a similar experiment in an isolated rat heart model (blood is absent) and found even higher DNA hypermethylation in cardiac tissue (by 9%), indicating the influence of circulatory molecules in the process of cardiac tissue hypermethylation. (5) Owing to a similar response of methylation pattern toward I/R in both blood and heart, the change in the methylation pattern of PBMC during I/R may provide insight into DNA methylation changes in cardiac tissue and subsequent I/R associated methylation linked pathology.

The epigenetic process like DNA methylation contributes to the cellular metabolic homeostasis in response to the environmental changes in the tissue, to get acclimatized. During I/R, cellular level oxygen, pH and osmolality will drastically change from the ischemic tissue and contribute to the phenotypic variations of different gene products (2). Early studies have shown that TET2 is an oxygen-dependent demethylating enzyme and its knockout is reported to promote DNA hypermethylation (27). Similarly, previous studies have demonstrated that overexpression of DNMT1 can induce genomic hypermethylation (28). In the present study, we noted low expression of TET 2 gene expression, an upregulation of the DNMT1 gene, and subsequent elevation in DNA methylation in cardiac tissue and PBMC in I/R rat heart. Many studies have showed that DNMT1 inhibitors like 5-azacytidine, decitabine and GSK-3484862 could mediate global DNA demethylation (29). Accordingly, when we targeted DNA methylation using 5-azacytidine (pre-treatment for 15 alternate days) in I/R hearts, DNMT1 expression declined in relation with global DNA hypomethylation. Furthermore, we found that TET 2 was upregulated along with the low level of DNA methylation in I/R rat hearts (Figures 1A,C). However, no conclusive evidence exists in the literature that connects a specific reciprocal relationship between DNMT1 and TET 2 gene expression in I/R hearts.

Interestingly, pre-treatment with DNMT inhibitor for 15 alternate days not only reversed the DNA methylation pattern but also reduced the area of the infarction, inflammation and cardiac injury markers (Figures 2, 3), emphasizing the importance of DNA methylation in cardioprotection against I/R. This is in corroboration with a previous finding in isolated rat heart model, where acute treatment/preconditioning the hearts with 5-azacytidine (DNMT inhibitor) rendered a similar kind of cardioprotection against I/R (9). However, more studies are required in this direction to make a conclusive statement that ascertain the importance of acute/chronic DNA hypomethylation in the cardioprotective mechanism.

Early studies in cancer tissues had shown that global DNA methylation has a significant influence on the expression of apoptosis-associated genes, thereby involved in the cell death process (30). Accordingly, in the present study, targeting global DNA methylation reversed the expression of apoptotic genes CASP3, CASP7, CASP9, and PARP and inflammatory genes in the I/R heart to near-normal level, resulting in cardioprotection (Figures 5A,J).

Earlier studies have shown that cardiac ischemia induces genome-wide promoter region hypermethylation that suppresses the expression of oxidative metabolic genes associated with mitochondrial ETC (OXPHOS) enzymes, TCA cycle, and fatty acid β oxidative enzymes in the heart, that promote colliquative myocytolysis (characterized to have suppressed myofibrillar protein expression and reduced energetic demand) (6). This metabolic switch enable the transition from higher to lower energy demand, that helps the ischemia-affected myocardium to be viable. Accordingly, in the present study, I/R induced global DNA hypermethylation and subsequently suppressed PGC-1α, TFAM, and POLG genes along with mt-DNA encoded OXPHOS genes. PGC-1α is the transcriptional regulator of mitochondrial homeostasis, which regulates the OXPHOS and antioxidant defense mechanism by promoting the mitochondrial transcription factor TFAM. Numerous studies report a correlation between DNA methylation of the PGC-1α and TFAM promoter regions and their subsequent transcription with the mtDNA copy number in metabolic disease condition (31, 32). Mitochondrial copy number is also reported to be influenced by the methylation status of POLG, responsible for mtDNA replication and repair (33). These studies suggest that DNA methylation promotes the PGC-1α-driven mitochondrial biogenesis, POLG-driven mtDNA replication and TFAM-driven mitochondrial transcription resulting in reduced copy number. Accordingly, in the present study, the PGC-1α, TFAM, and POLG were significantly reduced during I/R pathology with a decline in mtDNA copy number (Figures 6A,B). Alterations in mtDNA copy number due to dysfunctional mitochondrial POLG and PGC-1α can induce loss of mitochondrial oxidative phosphorylation (OXPHOS) and mitochondrial ATP generation (34). Apart from nuclear DNA, mtDNA is also involved in the assembly of the bioenergetics system by encoding the core genes of OXPHOS, which are reported to be under the methylation of mtDNA (35). Hypermethylation in the D-loop region of mitochondria are reported in the literature to compromise the ETC function of mitochondria and their ATP levels (36). The present study is in accordance with the literature where hypermethylation of mtDNA was observed in I/R hearts with a corresponding decline in 7 bioenergetic genes resulting in declined ETC activities and ATP. Targeting DNA methylation via DNMT inhibition significantly improved the mtDNA copy number via upregulating PGC-1α, POLG, and TFAM and reversed the mtDNA methylation and bioenergetic gene expression to normal level during I/R (Figures 6, 7), strengthening its capability as an excellent therapeutic target. Moreover, the I/R-mediated oxidative stress and the declined antioxidant enzymes were also significantly improved upon targeting methylation (Figure 8).

To summarize, we demonstrated in the present study that targeting DNA methylation, improved the expression of genes involved in the mitochondrial function, antioxidant enzymes, anti-apoptotic protein and inflammatory mediators to near-normal levels in I/R rat heart at transcriptional level, which significantly reduced the I/R-linked cardiac injury. However, it is noteworthy to mention that transcription is a relatively slow process, translation of pre-existing mRNA networks might affect cardiac gene expression, thereby rapidly adapting the myocardium to stress, before the initiation of I/R-triggered transcription. In fact, a previous report by Doroudgar et al. (37), suggested that early translational control at the eukaryotic translation initiation factors eIF6 and eIF3 regulate the translation of mitochondrial proteins, in response to acute pathological stress. Also, inhibiting the mTOR-mediated regulation of protein synthesis can modify the expression of metabolic genes (37) and reduce the formation of the eIF4F translation initiation complex, resulting in cardioprotection against I/R. Earlier studies have showed that the gene involved in translation initiation factor eIF3 is also regulated by DNA methylation at cg14297023 site of eIF3D subunit (38). Thus, DNA hypomethylation could possibly reduce the energy-consuming protein translation process by regulating the necessary protein translation machinery. Improved understanding of translational control could also result in novel therapeutic avenues based on specific translational inhibition alongside DNA methylation inhibition.

Despite the ischemia-induced changes in the heart, the peripheral tissue blood is also reported to undergo methylation and gene expression changes in ischemic heart disease patients (39). In fact, blood leukocyte DNA methylation was used in the early studies to predict future myocardial infarction and coronary artery diseases (40). PBMCs display both innate and adaptive immunity, experienced due to the I/R-mediated altered environment. The altered cellular environment imparts distinct disease-specific epigenetic signatures especially DNA methylation via Toll-like receptor ligands (41). TLR signaling plays a key role in innate immune defenses, thereby contributing significantly to cardiac ischemia/reperfusion injury. In the present study, we showed the elevation in DNA methylation in I/R rat PBMC sample and subsequent upregulation of DNMT1 gene expression (Figures 1B,D). In fact, the methylation changes were comparable between cardiac tissue and PBMC, prompting us to find if the blood contributes to the DNA methylation change in the heart. In this direction, we repeated the experiment in isolated rat heart model, where the heart was perfused with KH buffer rather blood, where we found a similar pattern of global DNA hypermethylation in I/R heart. Still, the extent of increase was greater in the I/R heart in the absence of blood (Figure 2A). However, the similarity between the myocardium and blood, in terms of global DNA methylation, and the expression of inflammatory cytokines and injury markers genes during the I/R pathology, in both the presence and absence of DNMT1 inhibitor suggest that the I/R mediators that are available in the two compartments are partially similar and blood can be utilized to predict the level of I/R-linked mediators in heart, especially the global DNA methylation. But whether blood can regulate the DNA methylation process in the myocardium is not conclusively established.

Considering the fact that blood samples are easy to collect and access, blood assessment under cardiac I/R settings to measure DNA methylation makes it convenient and used as a prediction tool in clinical setups for I/R injury. In fact, according to Xia et al. (39), blood methylation levels were reported to be biologically relevant to cardiac ischemic tissue, since blood leukocytes are responsible for the inflammation reaction in the heart, which initiates the formation of atheroma. However, whether blood-based DNA methylation and correspondingly altered genes accurately substitute for those obtained directly from the I/R tissue remains. Interestingly, a study by Liew et al. (42) showed that human blood was shown to express the heart-specific β-MHC transcript. Similarly, Adachi et al. (43) reported that the heart-specific miR-499 is upregulated in the plasma of myocardial infarction patients In line with these studies, the present study also showed that DNA hypermethylation and upregulation in the gene expression of dysferlin and inflammatory cytokines were similar to the heart-specific modifications during I/R challenge. In fact, Pearson’s correlation study between PBMC methylation and cardiac methylation showed a significant positive correlation between the two variables. However, further studies are required in this direction to support the DNA methylation and associated changes in gene expression of whole blood as a representative for the development of I/R in cardiac tissues.

## Data availability statement

The data presented in this study are available here: doi: 10.5281/zenodo.7052594.

## Ethics statement

This animal study was reviewed and approved by Institutional Animal Ethics Committee (IAEC), SASTRA Deemed University.

## Author contributions

GK designed the study, interpreted the data, and drafted the manuscript. SB performed the experiments, analyzed and interpreted the data, and drafted the manuscript. AA and NA helped with study design, literature review, and manuscript preparation. RK consulted in the design and interpretation of the data, and critically reviewed the manuscript. All authors proofread the manuscript.
